# Comprehensive analyses of m6A RNA methylation patterns and related immune microenvironment in idiopathic pulmonary arterial hypertension

**DOI:** 10.3389/fgene.2023.1222368

**Published:** 2023-09-04

**Authors:** Gufeng Gao, Ai Chen, Jin Gong, Weijun Lin, Weibin Wu, Sagor Mohammad Ismail Hajary, Guili Lian, Li Luo, Liangdi Xie

**Affiliations:** ^1^ Department of Geriatrics, The First Affiliated Hospital of Fujian Medical University, Fujian, Fuzhou, China; ^2^ Fujian Hypertension Research Institute, The First Affiliated Hospital of Fujian Medical University, Fujian, Fuzhou, China; ^3^ Clinical Research Center for Geriatric Hypertension Disease of Fujian Province, The First Affiliated Hospital of Fujian Medical University, Fujian, Fuzhou, China; ^4^ Branch of National Clinical Research Center for Aging and Medicine, The First Affiliated Hospital of Fujian Medical University, Fuzhou, Fujian, China; ^5^ Department of Geriatrics, National Regional Medical Center, Binhai Campus of the First Affiliated Hospital, Fujian Medical University, Fujian, Fuzhou, China

**Keywords:** idiopathic pulmonary arterial hypertension, m6A methylation regulators, immune microenvironment, human pulmonary artery smooth muscle cells, platelet-derived growth factor-BB

## Abstract

Idiopathic pulmonary arterial hypertension (IPAH) is a life-threatening disease with a poor prognosis and high heritability, characterized by elevated pulmonary vascular resistance (PVR) and pulmonary artery pressure. N6-methyladenosine (m6A) RNA modification influences many RNA metabolism pathways. However, the position of m6A methylation regulators in IPAH remains unknown. Therefore, the study aims to disclose the function m6A regulators exert in the pathological mechanisms of IPAH and the immune microenvironment involved. The GSE117261 dataset was downloaded from the Gene Expression Omnibus (GEO) to screen the differentially expressed genes (DEGs) between normal and IPAH samples. Functional and pathway enrichment analyses of DEGs were then conducted by Gene ontology (GO) analysis and Kyoto Encyclopedia of Genes and Genomes (KEGG). We also identified the differentially-expressed m6A (DEm6A) regulators between normal and IPAH samples. Key m6A regulators related to the prediction of IPAH were selected using the random forest model. The results showed that FMR1, RBM15, HNRNPA2B1 and IGFBP3 were upregulated in IPAH. In contrast, LRPPRC was downregulated. The single sample gene set enrichment analysis (ssGSEA) method was then adopted to estimate the immune microenvironment in distinct m6A clusters and m6A phenotype-related genes (PRGs) clusters, respectively. Furthermore, we calculated the m6A score via principal component analysis (PCA), and the Sankey diagram was selected to present the correlation among the m6A clusters, m6A PRGs clusters and m6A score. Finally, quantitative RT-PCR and Western blotting were used to validate the key genes in human pulmonary artery smooth muscle cells (HPASMCs) treated by human platelet-derived growth factor-BB (PDGF-BB). The relative mRNA and protein expression levels of FMR1 were significantly elevated, however, the relative mRNA and protein expression levels of LRPPRC were downregulated. Besides, the relative mRNA level of HNRNPA2B1 was increased. Generally, this bioinformatics analysis might provoke more insights into diagnosing and treating IPAH.

## 1 Introduction

Pulmonary arterial hypertension (PAH) is a rare progressive disease characterized by the occlusion of small pulmonary arteries due to endothelial dysfunction and excessive proliferation of pulmonary arterial smooth muscle cells (PASMCs) and fibroblasts. Treated ineffectively, patients with PAH will die of right heart failure (Aulak et al., 2021). However, almost 30%–40% of PAH patients could not be detected with definite risk factors or causes, so-called idiopathic pulmonary arterial hypertension (IPAH) (Kennedy and Rodgers, 2019). Meanwhile, IPAH is a life-threatening disease with a poor prognosis and high heritability, characterized by elevated pulmonary vascular resistance (PVR) and pulmonary artery pressure (Miyamoto et al., 2021). Despite numerous research on the occurrence and development of IPAH, prevention and treatment remain unsolved problems. Thus, exploring promising biomarkers and prognostic indicators is of obvious necessity for suppressing IPAH.

N6-methyladenosine (m6A) RNA modification, as the major type of RNA modification pattern in eukaryotes, influences many RNA metabolism pathways (Sun et al., 2019). In recent years, new forms of RNA methylations (m5C, m6A, m7G, m1A) have been revealed to occupy an important position in cardiovascular diseases, including pulmonary hypertension (PH), heart failure, hypertension, etc. (Zhou et al., 2021). Zeng et al., (2021). Proved that m6A levels and the expression of methylation-related enzymes were altered in PAH rats, playing crucial roles in PAH development (Zeng et al., 2021). In addition, one of m6A regulators, YTHDF1, has been confirmed to facilitate hypoxic PASMCs proliferation in mice. While silencing of YTHDF1 attenuated pulmonary vascular changes, pulmonary fibrosis and decreased right ventricular systolic pressure compared with PAH mice (Kang et al., 2023). However, the function of m6A regulators in the occurrence of IPAH remains unknown. Therefore, there is of great essential to identify m6A regulators in the development of IPAH and discover new predicted biomarkers and therapeutic targets for IPAH.

In this study, we performed functional and pathway enrichment analysis of differentially expressed genes (DEGs) between normal and IPAH lung tissues on the basis of Gene Expression Omnibus (GEO) databases. A total of 22 m6A regulators in IPAH were then analyzed, and the samples were collected to illustrate m6A modification patterns. Correlation analysis was applied between the m6A modification patterns and the immune microenvironment. Besides, m6A phenotype-related genes (PRGs) between distinct m6A modification patterns were used to generate the m6A PRGs signature. Finally, we validated the relative mRNA and protein expression levels of key genes in platelet-derived growth factor-BB (PDGF-BB) treated human pulmonary artery smooth muscle cells (HPASMCs). Our research established the first integrated bioinformatics analysis of the function of m6A regulators in IPAH, providing some valuable insights into the molecular mechanisms of IPAH at the biological level.

## 2 Materials and methods

### 2.1 Data collection and process

GEO is a public database providing high-throughput gene expression and genomics data sets (Barrett et al., 2013). The gene expression dataset GSE117261 (GPL6244, human lung tissues) (Stearman et al., 2019; Romanoski et al., 2020) was accessed from the GEO database and processed by R software (version 4.2.1) and Perl language. The GSE117261 dataset was based on the GPL6244 platform, and the sample species was *homo sapiens*. GSE117261 included 32 IPAH and 25 normal lung tissues. Genes with different expression levels between normal and IPAH lung tissues were collected via the “limma” package with the criterion of adjusted *p*-value <0.05 and |logFC| >1. A total of 22 m6A regulators were obtained from previous research to observe their expressions between IPAH and normal samples in GSE117261. The regulators include eight writers (METTL3, METTL14, METTL16, WTAP, ZC3H13, RBM15, RBM15B and CBLL1), twelve readers (YTHDC1, YTHDC2, YTHDF1, YTHDF2, YTHDF3, FMR1, LRPPRC, HNRNPA2B1, IGFBP3, IGFBP2, IGFBP3, and ELAVL1) and two erasers (FTO and ALKBH5).

### 2.2 Functional and enrichment analyses

Gene ontology (GO) analysis and Kyoto Encyclopedia of Genes and Genomes (KEGG) were then performed as functional and enrichment analyses based on the DEGs (adjusted *p*-value <0.05 and |logFC| >1). GO analysis, including biological processes (BPs), molecular functions (MFs) and cellular components (CCs), is a bioinformatics tool providing annotated genes and analysis of the gene products (Ashburner et al., 2000). Meanwhile, KEGG is a database resource with higher-order functional information for understanding comprehensive analysis of gene functions and genomic information (Kanehisa and Goto, 2000).

### 2.3 Construction of random forest

With *p* < 0.05, differentially-expressed m6A (DEm6A) methylation regulators between normal and IPAH tissues were selected through the “limma” package. The random forest (RF) and support vector machine (SVM) were both conducted to predict the occurrence of IPAH as training models. A receiver operating characteristic (ROC) curve, reverse cumulative distribution of residual and boxplots of residual were adapted to estimate effective models. Predicting the occurrence of IPAH based on the DEm6A methylation regulators was performed by RF with the “randomForest” package in R software.

### 2.4 Nomogram model

We established a nomogram model to screen candidate genes using the “rms” package. Calibration curves were constructed to compare the heterogenicity between the observed and the predicted values. Then, we used decision curve analysis (DCA) (Fu J. et al., 2022) and a clinical impact curve to validate the clinical effectiveness of nomograms.

### 2.5 Discover different m6A clusters and immune microenvironment involved

The “ConsensusClusterPlus” package was operated to differentiate the m6A clusters on the basis of DEm6A methylation regulators. The number of clusters was determined following the cumulative distribution function (CDF) curve and specific k values (Wilkerson and Hayes, 2010). Then, the m6A modification patterns were further validated using principal component analysis (PCA) (Fu et al., 2022). The single sample gene set enrichment analysis (ssGSEA), as a method to evaluate the number of distinct infiltrating immunocytes and their specific immune reactions, was then adapted to estimate the abundance of 23 immune cells between distinct m6A clusters to explore the correlation (Zhang et al., 2021).

### 2.6 Exploring m6A PRGs signature and immune microenvironment

Genes with different expression levels between distinct m6A clusters (as known as m6A PRGs genes) were picked out through the “limma” package. The selection criteria were formulated as |logFC| >0.5 and the adjusted *p*-value <0.05. Then, m6A PRGs were used to build the m6A Gene signature using the “ConsensusClusterPlus” package. Samples were then classified as distinct m6A PRGs clusters according to the m6A gene signature. The number of m6A PRGs clusters should align with the number of m6A clusters. Similarly, ssGSEA was then performed to observe the link between distinct immune cells and m6A gene signature.

### 2.7 m6A score calculation

PCA method was applied to calculate the m6A score of each sample based on DEm6A methylation regulators between the normal and IPAH groups. The formula

$$m6A\;score=\Sigma \;\left(PC1i+PC2i\right)$$

was used to calculate m6A score, where PC1 stands for principal component 1, PC2 stands for principal component 2, and i stands for the m6A-related genes. Moreover, box plots were performed to understand the m6A score in the two clustering types. Finally, the Sankey diagram was selected to present the correlation among the m6A clusters, m6A PRGs clusters and m6A score.

### 2.8 Isolation and treatment of HPASMCs

Human pulmonary arteriole samples were obtained based on the protocol approved by the Medical Research and Clinical Technology Application Branch of the Ethics Committee of the First Affiliated Hospital of Fujian Medical University [Approval No. MRCTA, FMU (2001) 483 ECFAH]. These samples were collected from 10 male patients, aged 60 ± 11 years, who had part of their lung lobes removed due to emphysema or lung abscess in the Department of Thoracic Surgery of the First Affiliated Hospital of Fujian Medical University. All participants have understood and signed the written informed consent. Meanwhile, we determined where to take the pulmonary arteries based on the lung lobe to be resected during surgery. Sterile ophthalmic scissors were used to cut the pulmonary arteries of patients into small pieces. Then, small pieces of pulmonary arteries were cultured in DMEM/F12 containing 20% FBS in a humidified environment of 5% CO_2_ at 37°C (Wu et al., 2022). The medium was replaced by DMEM with 0.02% FBS and starved for 24 h when the cells were 70%–80% confluent. Treated with human PDGF-BB (Pepro Tech) (20 ng/mL) for 48 h, HPAMSCs were collected for the following testing.

### 2.9 RNA extraction and real-time quantitative RT-PCR

The Fast-Pure Cell/Tissue Total RNA Isolation KIT V2 (Vazyme, Nanjing, China) was used to extract the total RNA from HPASMCs. HiScript II Q RT SuperMix for qPCR (+gDNA wiper) (Vazyme, Nanjing, China) was selected to obtain cDNA. Finally, Quantitative RT-PCR was performed with ChamQ SYBR qPCR Master Mix in the LightCycler^®^ 96 System (Roche Diagnostics, Mannheim, Germany). The relative expression level of mRNA was calculated based on the 2^−ΔΔCT^ method. The primer pairs used in Quantitative RT-PCR were as follows: FMR1 F: 5′-ACT​TAC​GGC​AAA​TGT​GTG​CCA-3′; R: 5′-GCA​GAC​TCC​GAA​AGT​GCA​TGT-3′; LRPPRC F: 5′-GAT​TGC​CTG​CCG​ATT​GAA​CC-3′; R: 5′- TGA​AGC​CCT​TGA​TGT​GGG​TC-3′; RBM15 F: 5′-GCA​GTC​CAG​AAT​TGA​GCA​GTA​G-3′; R: 5′-TAC​CTC​GTC​TGT​CTC​TGA​TTG​G-3′; HNRNPA2B1 F: 5′-TGG​TGG​TAG​CAG​GAA​CAT​GG-3′; R: 5′-TCA​GTA​TCG​GCT​CCT​CCC​AC-3′; IGFBP3 F: 5′-GAA​TCA​CCT​GAA​GTT​CCT​CAA​TGT-3′; R: 5′-CTT​ATC​CAC​ACA​CCA​GCA​GAA​G-3′; GAPDH F: 5′-GGT​GTG​AAC​CAT​GAG​AAG​TAT​GA-3′; R: 5′-GAG​TCC​TTC​CAC​GAT​ACC​AAA​G-3′.

### 2.10 Protein extraction and Western blotting

Western blotting was performed as previously described (Chen et al., 2010). After PDGF-BB administration, proteins from HPASMCs were extracted by lysis buffer. Lysates were collected to detect the protein concentrations using a BCA protein assay kit (Beyotime, China). Equal volumes of protein lysates were separated by 8% or 10% sodium dodecyl sulfate-polyacrylamide gel electrophoresis and electroblotted onto PVDF membranes before blocking with 5% nonfat milk for 1 h. Subsequently, blots were incubated in primary anti-FMR1 (1:500; Abcam), anti-LRPPRC (1:500; Proteintech), anti-RBM15 (1:500; Proteintech), antibodies at 4°C overnight. Then, the corresponding secondary antibody was diluted with the PVDF membrane for 1 h at room temperature after washing with TBST three times for 10 min each. Next, the blots were visualized using an ECL detection system (Beyotime, China). Further analysis of the blots was performed using ImageJ software (National Institutes of Health, Bethesda, MD, United States) (Zhong et al., 2018).

### 2.11 Statistical analysis

Perl, R for mac 4.2.1 software and R studio 2022.07.1 + 554 were conducted in our research. Student’s *t*-test or non-parametric one-way analysis of variance (ANOVA) was applied in a two-group comparison or three or more group comparisons, respectively. *p*-value of less than 0.05 with 2-sided defined statistically significant. **** means *p*-value <0.0001; *** means *p*-value <0.001; ** means *p*-value <0.01; * means *p*-value <0.05 and ns means no significance.

## 3 Results

### 3.1 Functional and pathway enrichment analysis

**FIGURE 1:**
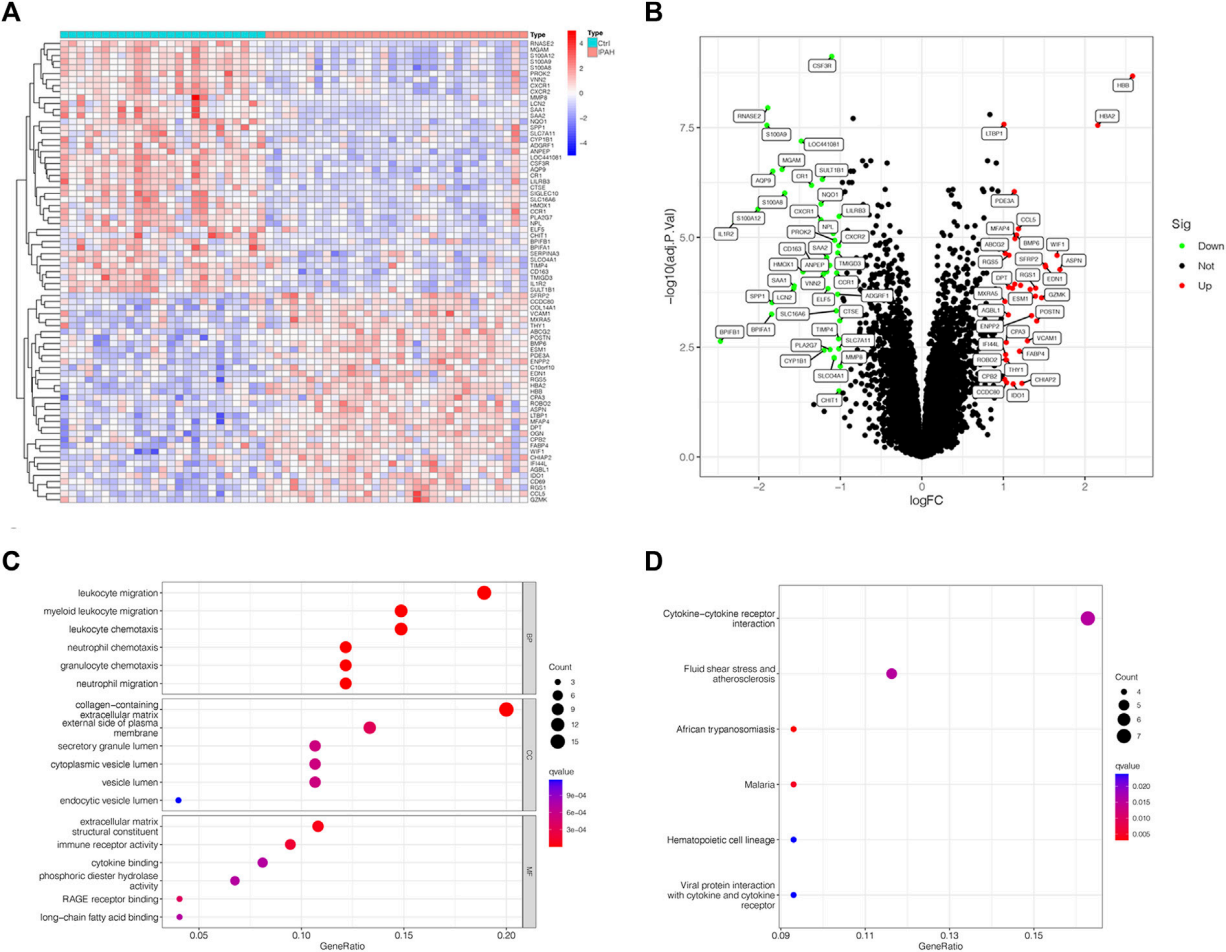
Functional and pathway enrichment analysis. **(A)** Heatmap of identified DEGs in lung tissues of normal and IPAH patients based on GSE117261 datasets. **(B)** A volcano map of the DEGs in 25 normal and 32 IPAH samples. **(C)** The BP, CC and MF of DEGs. **(D)** KEGG analysis illustrating pathway enrichment of DEG. DEG, differentially-expressed genes.

DEGs between normal and IPAH samples were collected with the criteria of adjusted *p*-value <0.05 and |logFC| >1. A heatmap was conducted to show the DEGs expression in each sample (Figure 1A). Meanwhile, a volcano map was displayed for an in-depth understanding of the DEGs (Figure 1B). Functional and pathway enrichment analyses were subsequently applied to understand 77 DEGs between normal and IPAH samples comprehensively. The top 6 BP, CC and MF results according to the *p*-value were listed in Figure 1C. Meanwhile, the results of KEGG enrichment analysis suggested that DEGs were statistically concentrated in Pathways of Fluid shear stress and atherosclerosis, Cytokine-cytokine receptor interaction, African trypanosomiasis, cytokine receptor, Malaria and Viral protein interaction with cytokine and Hematopoietic cell lineage (Figure 1D). Notably, MF results showed that immune receptor activity changed significantly, indicating the disordered immune activity involved in the IPAH development.

### 3.2 The outlook of m6A regulators in the lung tissues of normal and IPAH samples

**FIGURE 2:**
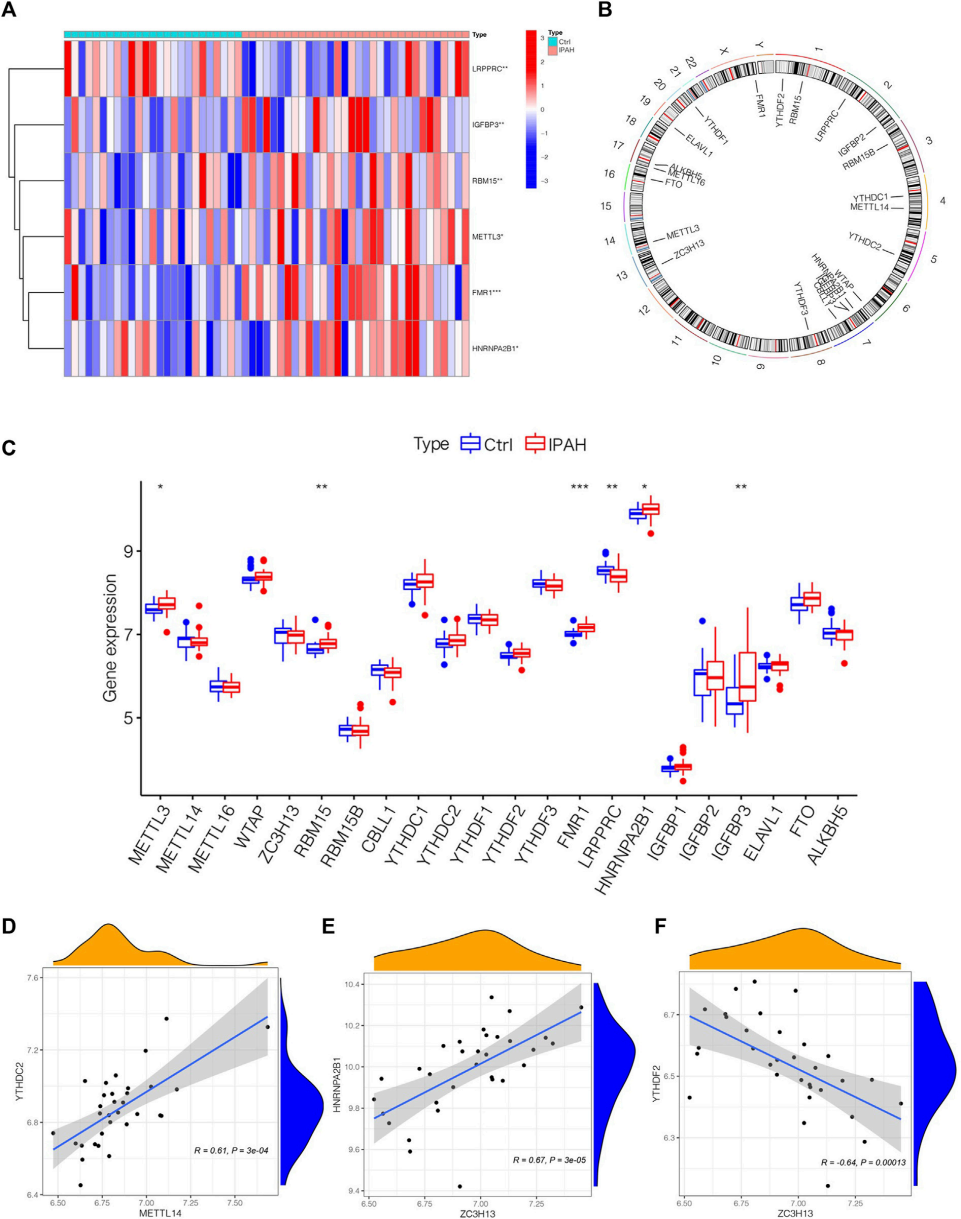
The outlook of m6A regulators in the lung tissues of normal and IPAH samples. **(A)** Heatmap of significantly differential-expressed 6 m6A regulators in samples of GSE117261 datasets. **(B)** The location of different m6A regulators on chromosomes. **(C)** Boxplot displaying m6A regulators of different expression levels between Ctrl and IPAH samples. *p < 0.05, **p < 0.01, ***p < 0.001. **(D)** The association between the expression level of METTL14 and YTHDC2. **(E)** The associations between the expression level of ZC3H13 and HNRNPA2B1. **(F)** The associations between the expression level of ZC3H13 and YTHDF2. Ctrl, normal group; IPAH, idiopathic pulmonary arterial hypertension.

To depict the landscape of m6A methylation regulators, we analyzed the differential m6A methylation regulators between normal and IPAH lung tissues. The GSE117261 dataset was used to perform the subsequent analysis. After exploring DEm6A methylation regulators in control (*n* = 25) and IPAH (*n* = 32), the expression of m6A methylation regulators was shown by a heatmap (Figure 2A). We also analyzed the location of 22 m6A regulators on chromosomes (Figure 2B). Precisely, the expression of FMR1, RBM15, HNRNPA2B1, IGFBP3 and METTL3 were elevated in IPAH (the criterion of adjusted *p*-value <0.05 and |logFC| >1); however, LRPPRC was downregulated (the criterion of adjusted *p*-value <0.05 and |logFC| >1) in IPAH compared with the normal samples (Figure 2C). Furthermore, a co-expression analysis was performed to indicate the positive correlation between METTL14 and YTHDC2, ZC3H13 and HNRNPA2B1, respectively (Figures 2D, E), and the negative correlation between ZC3H13 and YTHDF2 (Figure 2F). In short, 6 differential m6A methylation regulators (FMR1, RBM15, HNRNPA2B1, IGFBP3, METTL3 and LRPPRC) were screened for subsequent studies with the criterion of adjusted *p*-value <0.05 and |logFC| >1.

### 3.3 The RF was selected to build the IPAH predictive models

**FIGURE 3:**
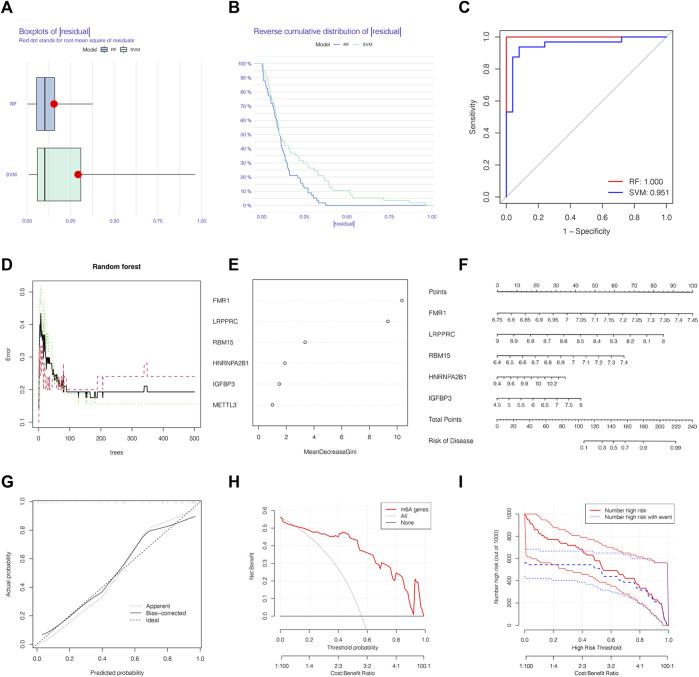
The RF was selected to build the IPAH predictive models. **(A)** Residual distribution of RF and SVM displayed by a boxplot. **(B)** The reverse cumulative distribution of residual curve of RF and SVM model. **(C)** The prediction ability of SVM and RF models shown by ROC curves. **(D)** The prediction error curves in the RF model. **(E)** Ranking of differential-expressed m6A regulators by their importance according to RF. **(F)** Nomogram model based on five m6A regulator genes to detect the predictive ability. **(G)** The production efficiency nomogram model illustrated by the calibration curves. **(H,I)** Determination of clinical prediction validity of nomogram model based on decision curve analysis and clinical impact plot. RF, random forest; Ctrl, normal group; IPAH, idiopathic pulmonary arterial hypertension.

We chose boxplots of residual, reverse cumulative distribution of residual, and a ROC curve (Figures 3A–C) to appraise the prediction accuracy of the RF and SVM methods. Obviously, the RF method is of higher prediction accuracy, so RF method was chosen for ranking the importance of the DEm6A regulators. The optimal ntree was confirmed in Figure 3D. Then, the top 5 genes were defined as the 5 most significant m6A methylation regulators after ranking their importance (Figure 3E). Then, a nomogram model was applied to estimate the susceptibility to IPAH according to the 5 m6A methylation regulators (Figure 3F). The calibration curves revealed that the nomogram method has high predictive power for IPAH (Figure 3G). Finally, decision curve analysis (DCA) and clinical impact curves manifested that the nomogram model was a reliable predictive model for IPAH (Figures 3H, I). It is noteworthy that FMR1, LRPPRC, RBM15, HNRNPA2B1and IGFBP3 were selected as 5 most crucial m6A methylation regulators based on RF method to construct the nomogram model for IPAH.

### 3.4 The link between distinct m6A clusters and immune microenvironment

**FIGURE 4:**
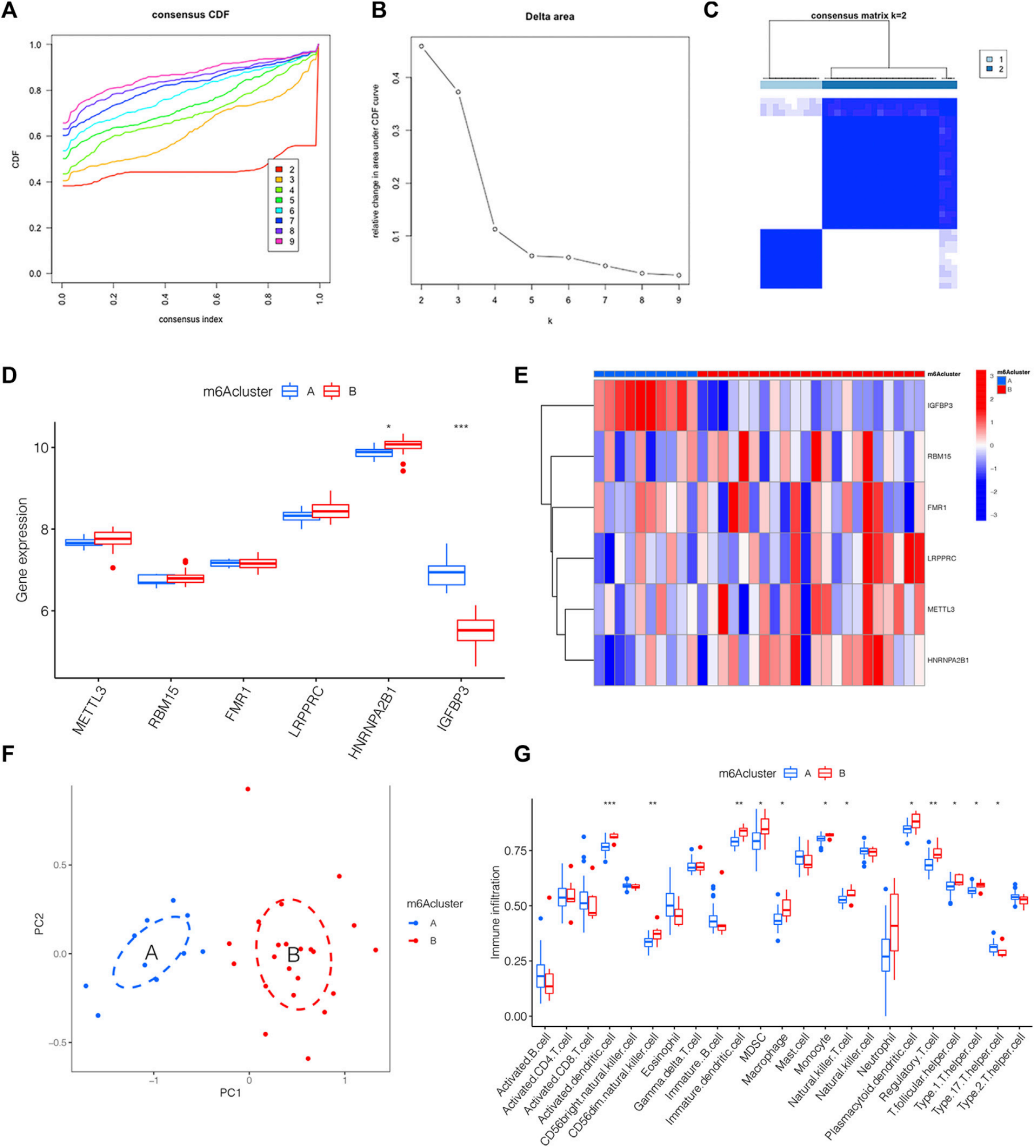
The link between distinct m6A clusters and immune microenvironment. **(A)** The consensus CDF curve based on various k (k = 2–9). **(B)** Relative change in area under distinct CDF curve (k = 2–9). **(C)** Consensus clustering matrix of 32 IPAH patients for k = 2. **(D)** The expression level of 6 m6A methylation regulator genes between distinct m6A clusters shown by the boxplot. **(E)** Heatmap displaying the transcriptional profile of 6 m6A methylation regulator genes of distinct m6A clusters. **(F)** PCA illustrating a significant difference between m6A clusters A and B. **(G)** Boxplot presenting the immune microenvironment in m6A clusters A and B. *p < 0.05, **p < 0.01, ***p < 0.001.

We classified distinct m6A clusters according to the seven m6A methylation regulators by conducting the “ConsensusClusterPlus” package in R. Taken 2 as the optimal k value, 32 IPAH samples were grouped into m6A clusters A and B (Figures 4A–C). Compared with m6A cluster A, the expression level of HNRNPA2B1 was higher in m6A cluster B, whereas IGFBP3 declined (Figure 4D). The heat map was also used to illustrate the distinct expression profiles of genes in two m6A clusters (Figure 4E). Then, PCA was used to validate the accuracy of our m6A cluster classification (Figure 4F). Considering the link between IPAH and the immune microenvironment, a deeper analysis of immune cell infiltration was applied. A total of 12 types of immune cells were determined to be statistically different between m6A clusters A and B in Figure 4G, implying that the immune response differs between the two clusters.

### 3.5 The link between m6A methylation regulators and immune microenvironment

**FIGURE 5:**
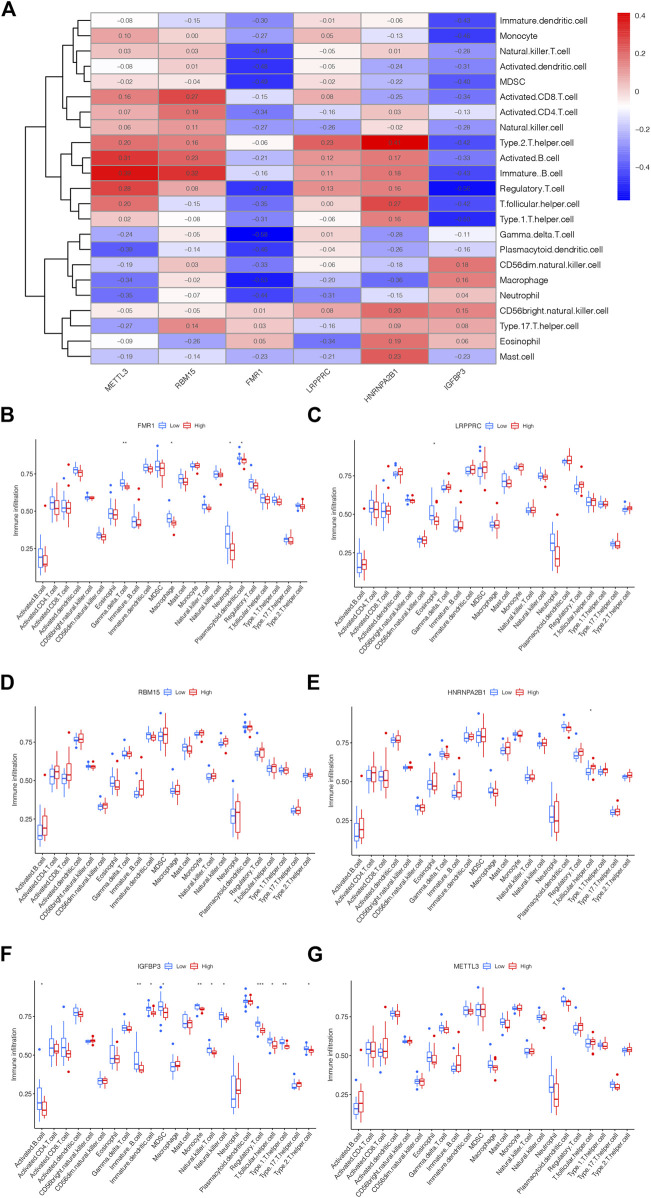
The link between m6A methylation regulators and immune microenvironment. **(A)** Heatmap showing the correlation between m5C methylation regulator genes and immune microenvironment. **(B)** The correlation between FMR1 and immune microenvironment. **(C)** The correlation between LRPPRC and immune microenvironment. **(D)** The correlation between RBM15 and immune microenvironment. **(E)** The correlation between HNRNPA2B1 and immune microenvironment. **(F)** The correlation between IGFBP3 and immune microenvironment. **(G)** The correlation between METTL3 and immune microenvironment. *p < 0.05, **p < 0.01, ***p < 0.001.

The link between immune cell infiltration and the six m6A methylation regulators’ expression profile was demonstrated via a heatmap based on ssGSEA (Figure 5A). Furthermore, we explored the relationship between the immune cell infiltration and six m6A methylation regulators via ssGSEA. As shown in Figure 5B, FMR1 was negatively correlated with Macrophage, Gamma delta T cell, Plasmacytoid dendritic cell and Neutrophil; LRPPRC was negatively correlated with Eosinophil as displayed in Figure 5C; The levels of RBM15 and METTL3 were not significantly linked with immune cell infiltration (Figures 5D, G); HNRNPA2B1 was positively linked with T follicular helper cell could be drawn from Figure 5E; while the IGFBP3 was negatively correlated with Activated B cell, Immature B cell, MDSC, Immature dendritic cell, Natural killer T cell, Natural killer cell, Monocyte, Regulatory T cell, T follicular helper cell, Type 1T helper cell and Type 2T helper cell, displayed in Figure 5F. The above findings confirmed the potential association of these DEm6A methylation regulators with the immune microenvironment in IPAH.

### 3.6 Generation of the m6A PRGs signature and immune microenvironment

**FIGURE 6:**
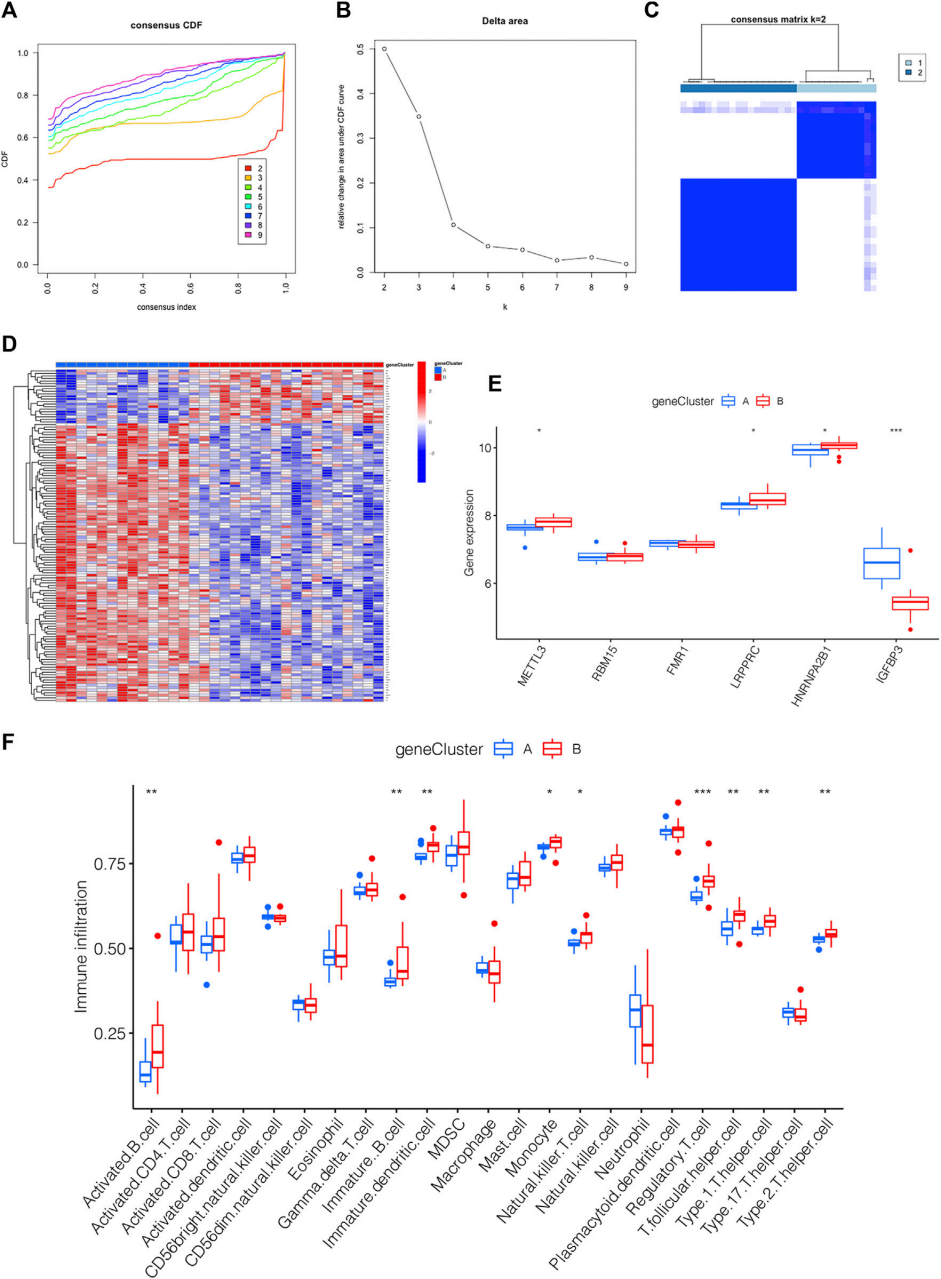
Generation of the m6A PRGs signature and immune microenvironment. **(A–C)** The consensus clustering of 130 m6A PRGs. **(D)** Heatmap showing the differential expression of m6A PRGs in distinct gene clusters. **(E)** Expression outlook of 6 m6A methylation regulators in distinct m6A PRGs clusters shown by a boxplot. **(F)** The immune microenvironment in the two m6A PRGs clusters. *p < 0.05, **p < 0.01, ***p < 0.001. Gene cluster, m6A PRGs clusters.

m6A PRGs signature was constructed on the basis of m6A PRGs through the “ConsensusClusterPlus” package in R. In order to be consistent with the number of m6A clusters, the optimal k value of m6A PRG clusters was set as 2 (Figures 6A–C). A heatmap displaying the different expression profiles of m6A PRGs between two m6A PRGs clusters was adopted (Figure 6D). Compared with m6A PRGs cluster A, the expression level of LRPPRC, HNRNPA2B1 and METTL3 were increased in m6A PRGs cluster B; however, IGFBP3 showed the opposite trend (Figure 6E). Finally, we found that nine types of immune cells were statistically differentially expressed between m6A PRGs clusters A and B (Figure 6F), indicating the distinguished characteristics of immune microenvironment infiltration.

### 3.7 m6A score determination

**FIGURE 7:**
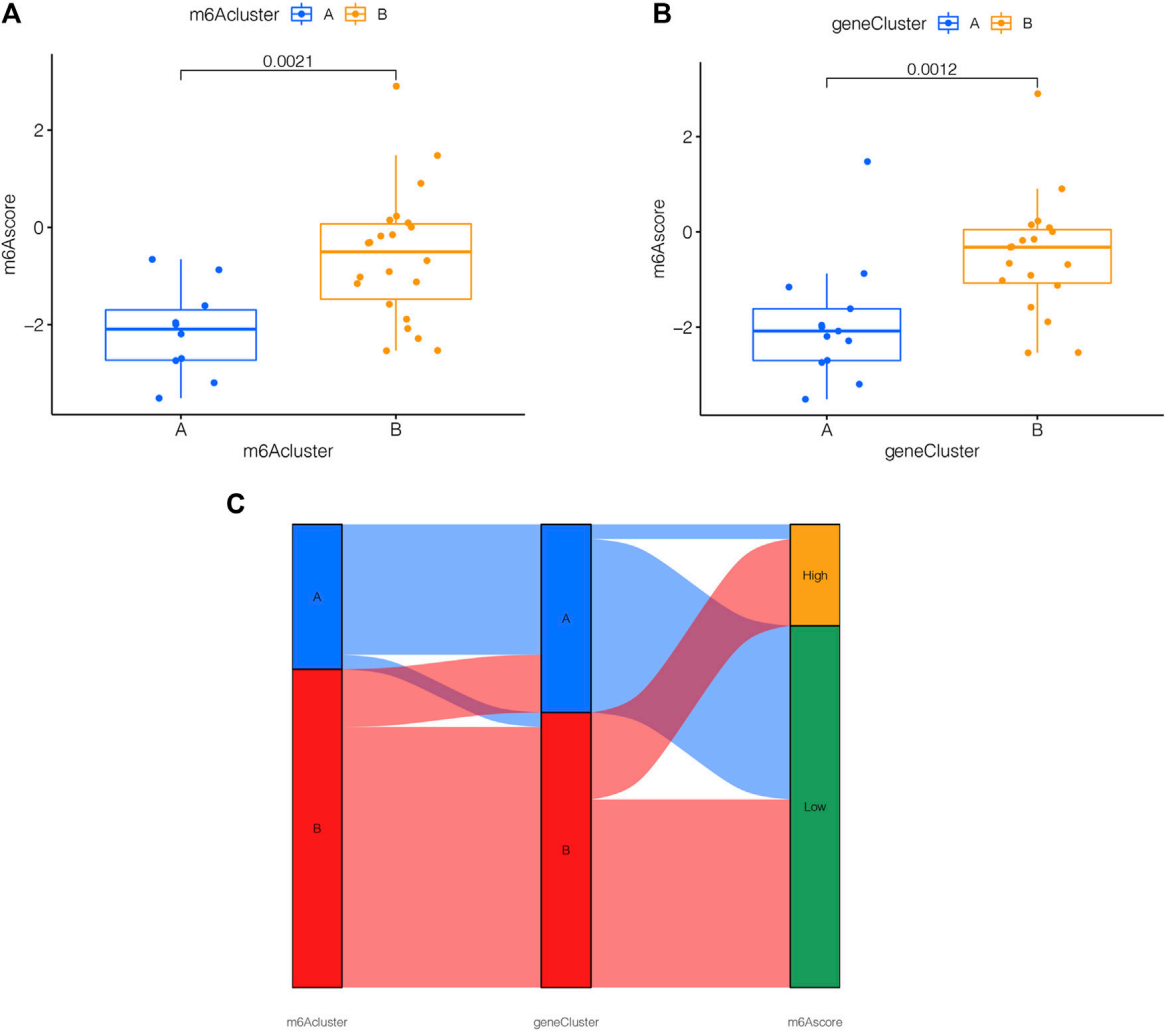
m6A score determination. m6A score calculated between **(A)** distinct m6A clusters and **(B)** m6A PRGs clusters. **(C)** Sankey diagram demonstrating the corresponding relations among m6A cluster, m6A PRGs clusters and m6A score. Gene cluster: m6A PRGs clusters.

For the sake of observing the different m6A score in m6A clusters and m6A PRGs clusters, boxplots were then applied, respectively. The m6A score of m6A cluster B was higher than that in m6A cluster A as shown in Figure 7A. Compared to m6A PRGs cluster A, m6A score was higher in m6A PRGs cluster B (Figure 7B). Thus, the m6A score in diverse m6A clusters and m6A PRGs clusters were both statistically different. To better understand the corresponding relations among m6A score, m6A clusters and m6A PRGs clusters, we used the “ggalluvial” R package to produce the Sankey diagram (Figure 7C).

### 3.8 The mRNA and protein expression levels of the key genes

**FIGURE 8:**
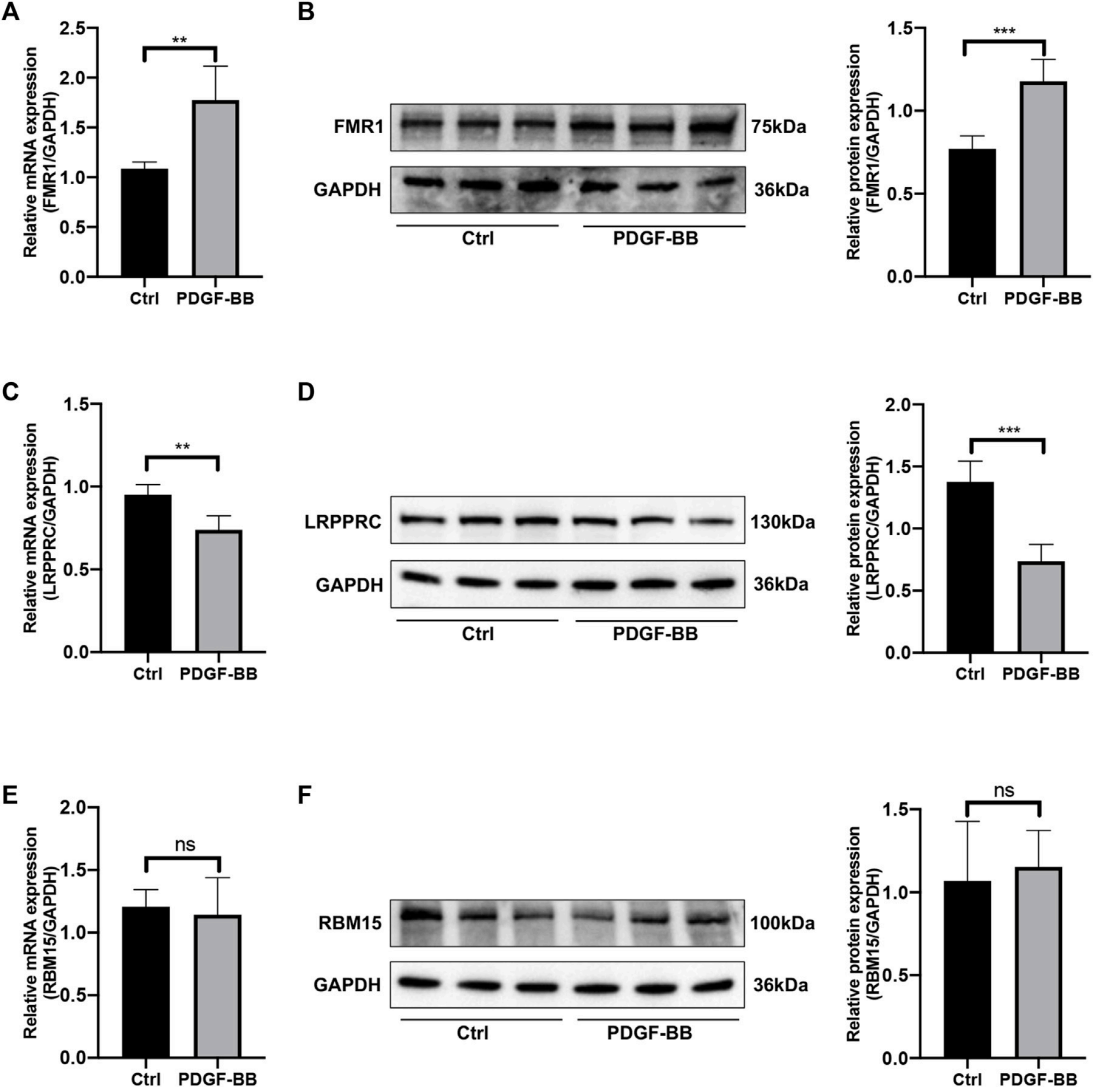
The mRNA and protein expression levels of key genes. **(A)** Relative mRNA level of FMR1 in control vs. PDGF-BB treated group. **(B)** Relative protein expression level of FMR1 in control vs. PDGF-BB treated group. **(C)** Relative mRNA level of LRPPRC in control vs. PDGF-BB treated group. **(D)** Relative protein expression level of LRPPRC in control vs. PDGF-BB treated group. **(E)** Relative mRNA level of RBM15 in control vs. PDGF-BB treated group. **(F)** Relative protein expression level of RBM15 in control vs. PDGF-BB treated group. *p < 0.05, **p < 0.01, ***p < 0.001, ns, no significance. Data are presented as mean ± standard deviation (mean ± SD), *n* = 5.

In order to estimate the expression levels of the selected key genes in HPASMCs with PDGF-BB administration, the quantitative RT-qPCR experiment and Western blotting were conducted. After being treated with PDGF-BB for 48 h, the mRNA and protein expression levels of FMR1 were upregulated (Figures 8A, B); however, the mRNA and protein expression levels of LRPPRC were downregulated in comparison with the control groups (Figures 8C, D), both were in line with the bioinformatics analysis. While the mRNA and protein expression levels of RBM15 remained unchanged (Figures 8E, F), which were not consistent with the results of bioinformatics analysis. Besides, the relative mRNA level of HNRNPA2B1 was increased; the relative mRNA level of IGFBP3 was not changed (Supplementary Figure S1).

## 4 Discussion

The IPAH, characterized by elevated PVR due to lung remodeling and/or vasoconstriction, is a serious disease leading to cardiopulmonary dysfunction and premature death (Barnes et al., 2019). Recently, new RNA methylations (m5C, m6A, m7G, m1A) have been demonstrated to be a promising target for improving CVDs, including PH (Zhou et al., 2021). As the most common RNA modification in eukaryotes, m6A levels and the expression of m6A methylation regulators have been reported to be altered in monocrotaline (MCT)-induced PAH rats by an integrated analysis (Zeng et al., 2021). Meanwhile, METTL3 and YTHDF2 were proven to be involved in the PASMCs proliferation induced by hypoxia, providing a novel insight for treating hypoxic PH (HPH) (Qin et al., 2021). However, the function of m6A methylation regulators in IPAH has not been discussed yet.

Therefore, in this research, bioinformatics analysis was adopted on the basis of the GSE117261 database. GO and KEGG were performed to analyze the functional and pathway enrichment based on the DEGs between 25 normal samples and 32 IPAH samples. The results of MF suggested that the pathology of IPAH may be related to abnormal immune activity. Previous reports have also shown that compromised immune homeostasis might lead to IPAH progression (Heukels et al., 2021).

Besides, 6 differential-expressed m6A methylation regulators were screened, including 5 upregulated regulators and 1 downregulated regulator. Compared with SVM, the RF showed higher accuracy in predicting disease occurrence. Thence, FMR1, LRPPRC, RBM15, HNRNPA2B1, and IGFBP3 were identified as the 5 most important genes after ranking their importance via the RF model. Among them, the expression level of FMR1, RBM15, HNRNPA2B1 and IGFBP3 were upregulated in IPAH; however, LRPPRC was downregulated. Meanwhile, FMR1 ranked as the most crucial one.

Studies in recent years have revealed similarities in the pathological mechanisms of PAH and cancer, including increased cell proliferation, resistance to apoptosis, and enhanced Warburg effect, suggesting PAH is a pseudo-malignant disease (Rehman and Archer, 2010; Boucherat et al., 2017; Liu et al., 2017). Besides, elevated expression of FMR1 was regarded as a pathogenic target in promoting cell proliferation in esophageal squamous cell carcinoma (ESCC) (Men et al., 2022); the positive feedback loop HNF4α-BC200-FMR1 is also necessary in promoting invasive mucinous lung adenocarcinoma (IMA) progression and metastasis. Based on the above research, we assumed that the increased FMR1 might be associated with the hyperproliferation of PASMCs.

LRPPRC, as a mitochondrion-associated protein, has been demonstrated as an autophagy inhibitor in many pieces of research. LRPPRC has been reported to decline under constant mitophagy stress, then initiating the autophagy level in cells by impairing the stability of Bcl-2 (Zou et al., 2013; Zou et al., 2014; Zou et al., 2015). In addition, increased autophagy level have been reported to play a significant role in many lung diseases, including PAH (Ornatowski et al., 2020). Thus, we supposed the decreased LRPPRC in our results might lead to enhanced autophagy levels in IPAH, which has not yet been illustrated in previous studies.

In our results, RBM15 is elevated. Consistent with previous research, increased RBM15 is also related to the development of tumors, such as colorectal cancer, laryngeal squamous cell cancer (LSCC) and pancreatic adenocarcinoma. It was reported that knocking down RBM15 inhibits colorectal cancer cell proliferation and metastasis as reported (Zhang Z. et al., 2021). Meanwhile, as a “writer” of methyltransferase, RBM15 was increased in LSCC, indicating an unfavorable prognosis; the decline of RBM15 reduced the proliferation, migration and invasion of LSCC cells (Wang et al., 2021). Besides, suppression RBM15 could significantly reduce pancreatic cancer cell proliferation in pancreatic adenocarcinoma (Zhao et al., 2022). Due to the similarity of PAH and tumors, we supposed RBM15 was a risk factor in the development of IPAH, which has not been proposed previously.

Similarly, increased HNRNPA2B1 was found in human PAH-PASMC and MCT-PAH rats, and inhibition of HNRNPA2B1 applied *in vivo* rescued PH in rats (Ruffenach et al., 2022); IGFBP-3 was revealed to promote the proliferation of HPASMCs under persistent hypoxia (Ismail et al., 2009); METTL3/YTHDF2/PTEN axis also plays a crucial role in hypoxia-induced PASMCs proliferation (Qin et al., 2021). In general, our analysis identified FMR1, LRPPRC and RBM15 as novel factors involved in PAH as well as IPAH for the first time, which is one of the most crucial scientific significances for our research. Therefore, FMR1, LRPPRC and RBM15 were selected for quantitative RT-PCR and Western blotting validation. While for HNRNPA2B1 and IGFBP3, only quantitative RT-PCR was performed.

Previous reports displayed that PASMCs from patients with IPAH showed a higher growth rate stimulated by PDGF-BB than that of control cells (Ogawa et al., 2005; Fujio et al., 2006; Ikeda et al., 2010); besides, the PDGF-receptor inhibitor such as imatinib (STI571) is attracting more and more attention as a promising therapy for PH (Ghofrani et al., 2005; Schermuly et al., 2005; Perros et al., 2008; Hatano et al., 2010). Therefore, we observed the relative mRNA and protein expression levels of key genes in HPASMCs treated by PDGF-BB for 48 h.

Besides, the results of MF indicated that abnormal immune receptor activity was involved in IPAH. Furthermore, we found that the immune cells infiltration in two m6A clusters and two m6A PRGs clusters were identified, suggesting the distinguished immune responses in the different m6A clusters. We also used ssGSEA to estimate the relationship between the immune cell infiltration and six DEm6A methylation regulators. The results incarnate a tight correlation between the immune microenvironment in IPAH and these six methylation regulators. Besides, several past reports confirmed our fundings. Qu et al., (2022) demonstrated that PAH might be associated with dysregulation of the immune microenvironment as well as an abnormal immune response (Qu et al., 2022). In addition, modulation of the immune microenvironment may help to slow the progression of PH (Guo et al., 2021). The previous research also explored the potential association of m6A RNA methylation regulators with the tumor immune microenvironment in Esophageal squamous cell carcinoma (ESCC), indicating that these key m6A methylation regulators may be crucial mediators of immune cell infiltration (Guo et al., 2021). Similarly, it was shown that m6A-regulated genes are linked to immune status in hepatocellular carcinoma (HCC) (Li et al., 2022). In our result, the m6A score of m6A clusters and m6A PRGs clusters were also calculated to have a better understanding of samples in different clusters based on DEm6A methylation regulators.

Whereas, there are still some flaws in our research. First, the mechanisms underlying IPAH were still unclear; thus, deeper bioinformatics analyses and experimental verifications are needed. Second, multiple microarray analyses have higher detection accuracy than single microarray analyses, so integrated microarray analyses should be performed in a future study. Third, to improve the prediction accuracy of the screened genes related to IPAH, it is necessary to enlarge the sample size for further proof. Fourth, because of clinical data lacking and heterogeneity of IPAH patients, risk indicators related to the degree of patients under the severity of IPAH are hard to summarize. Fifth, samples of IPAH with different severity are essential to distinguish the mechanisms underlying the occurrence and development of IPAH.

In conclusion, our study provided an integrated analysis by processing GSE117261 to discover the DEm6A regulator genes connected to the development of IPAH. 77 DEGs between normal and IPAH samples were collected for functional and pathway enrichment analysis, indicating the abnormal immune activity involved with IPAH. 5 key m6A modification regulators were screened out. 2 m6A clusters and 2 m6A PRGs clusters were distinguished. Besides, the immune microenvironment correlated to different m6A modification patterns was also checked. Furthermore, quantitative RT-PCR and Western blotting were performed to determine the key genes in HPASMCs stimulated by PDGF-BB. The relative mRNA and protein expression levels of FMR1 were increased, while the relative mRNA and protein expression levels of LRPPRC were decreased. Besides, the relative mRNA level of HNRNPA2B1 was significantly increased.

## Data Availability

Publicly available datasets were analyzed in this study. This data can be found here: https://www.ncbi.nlm.nih.gov/geo/query/acc.cgi?acc=GSE117261.
