# Supplementary material for: Comprehensive analyses of m6A RNA methylation patterns and related immune microenvironment in idiopathic pulmonary arterial hypertension
Source: Front Genet. 2023 Sep 4;14:1222368. doi: 10.3389/fgene.2023.1222368 (PMC10507408; doi:10.3389/fgene.2023.1222368)
Supplement: Supplementary file 1 [file Image1.pdf]

Supplementary Material

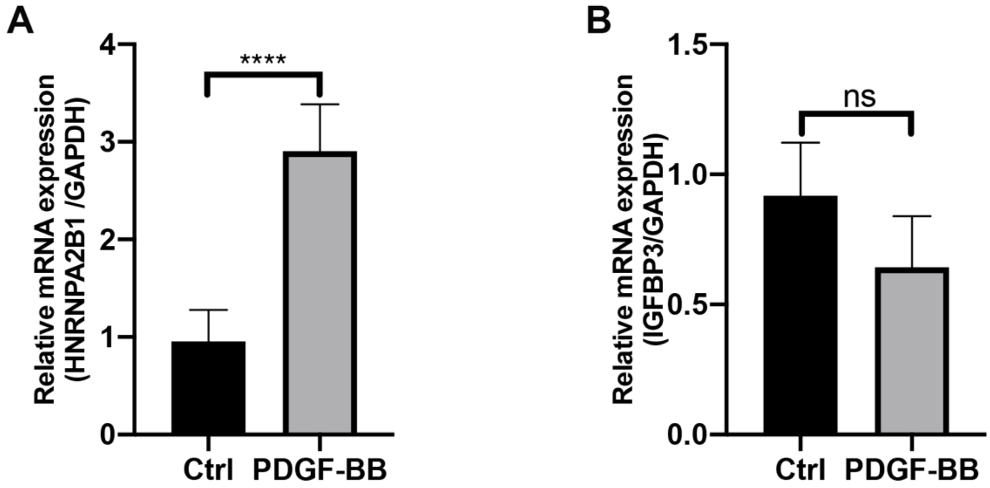

Supplementary Figure1: The mRNA levels of HNRNPA2B1 and IGFBP3. (A) Relative mRNA level of HNRNPA2B1 in control vs. PDGF-BB treated group. (B)Relative mRNA level of IGFBP3 in control vs. PDGF-BB treated group. *P<0.05, **P<0.01, ***P<0.001, ****P<0.0001, ns: no significance. Data are presented as mean ± standard deviation (mean ± SD), n = 5.
